# Trace Elemental Imaging of Rare Earth Elements Discriminates Tissues at Microscale in Flat Fossils

**DOI:** 10.1371/journal.pone.0086946

**Published:** 2014-01-29

**Authors:** Pierre Gueriau, Cristian Mocuta, Didier B. Dutheil, Serge X. Cohen, Dominique Thiaudière, The OT1 Consortium, Sylvain Charbonnier, Gaël Clément, Loïc Bertrand

**Affiliations:** 1 CR2P, UMR 7207 CNRS, MNHN, UPMC, Paris, France; 2 IPANEMA, USR 3461 CNRS, MCC, Gif-sur-Yvette, France; 3 Synchrotron SOLEIL, Gif-sur-Yvette, France; 4 15 passage du Buisson Saint-Louis, Paris, France; Argonne National Laboratory, United States of America

## Abstract

The interpretation of flattened fossils remains a major challenge due to compression of their complex anatomies during fossilization, making critical anatomical features invisible or hardly discernible. Key features are often hidden under greatly preserved decay prone tissues, or an unpreparable sedimentary matrix. A method offering access to such anatomical features is of paramount interest to resolve taxonomic affinities and to study fossils after a least possible invasive preparation. Unfortunately, the widely-used X-ray micro-computed tomography, for visualizing hidden or internal structures of a broad range of fossils, is generally inapplicable to flattened specimens, due to the very high differential absorbance in distinct directions. Here we show that synchrotron X-ray fluorescence spectral raster-scanning coupled to spectral decomposition or a much faster Kullback-Leibler divergence based statistical analysis provides microscale visualization of tissues. We imaged exceptionally well-preserved fossils from the Late Cretaceous without needing any prior delicate preparation. The contrasting elemental distributions greatly improved the discrimination of skeletal elements material from both the sedimentary matrix and fossilized soft tissues. Aside content in alkaline earth elements and phosphorus, a critical parameter for tissue discrimination is the distinct amounts of rare earth elements. Local quantification of rare earths may open new avenues for fossil description but also in paleoenvironmental and taphonomical studies.

## Introduction

Soft-bodied organisms normally degrade too fast to become fossilized. However, such fossils have been conserved in an exceptional preservation state over millions of years in specific deposits called Konservat-Lagerstätten [1]. Those contexts allowed high quality preservation of decay prone tissues or fully articulated skeletons and thus are of crucial importance to understand the biosphere and evolutionary history of the Earth [2]. However, the interpretation of soft-bodied fossils from Lagerstätten remains particularly challenging because most of them have been flattened during fossilization. The attending visual superimposition of anatomical features renders important characters either hardly distinguishable from each other, or hidden under other tissues [3].

For the past twenty years, paleontologists have used and tested point spectroscopy and mapping to analyze the elemental composition of flattened fossils. Electron microprobes and energy- or wavelength-dispersive X-ray spectrometry were both used on polished blocks, thin sections or unprepared fossil material preserved essentially in two dimensions [4]–[7]. Scanning electron microscopy (SEM) can reveal surface anatomical details that are not evident under light microscopy. Elemental maps by means of SEM energy-dispersive X-ray spectroscopy (SEM-EDX) gave access to the morphology of Cambrian Burgess Shale animals [5]. Elemental detectability is in the range of 2–5 wt% for EDX systems [8]. Acquisition time for SEM-EDX elemental maps were typically one to a few hours to resolve detail on square millimeters at a sub-micrometer spatial resolution [4]. SEM-EDX is thus an effective technique for major and minor element surface analyses. Yet, prolonged exposure to an electron beam can damage organic or inorganic samples [9]. Laser ablation-inductively coupled plasma-mass spectrometry (LA-ICP-MS) was used for the microscale mapping of trace elements and particularly rare earth elements (REEs) in fossil bones with detection limits for REEs of *ca.* 0.1 parts per million (ppm) and spot diameter ranging from 5 to 300 µm [10]. LA-ICP-MS is based on the local ablation of the sample area under investigation with a laser beam. The depth of material ablated is function of the level of concentration in the elements of interest and can reach 10 µm or more for quantification of traces of transition metals in mineral matrices.

The recent advent of focused and full-field X-ray imaging techniques provides considerable additional information for paleontological studies. Synchrotron scanning transmission X-ray microscopy (STXM) appears as a promising sensitive tool to decipher organic signature from heavily diagenetized specimens on submicrometric thin sections of fossils [11]. X-ray micro-computed tomography, which allows imaging the internal structures of a large variety of fossils through 3D reconstructions, has become in the recent years a major non-destructive tool in many fields of paleontology [12]–[14]. However, it suffers from a physical limit for the study of flattened fossils due to the extremely high difference in X-ray absorbance in different directions. This limitation has only partly been solved using approaches such as laminography [15]. Recently, synchrotron-based X-ray fluorescence (XRF) was used to collect integrated intensity in preselected spectral regions of interest and to map metal distributions, particularly copper, in fossil bird feathers and in lizard skins with a spot diameter of 0.1 mm [16]–[19].

Here, we discriminate tissues in exceptionally well-preserved fossils on the basis of their content in chemical elements from majors to traces, in particular trace rare earths and transition metals, and alkaline earths. We exploit the distinct affinities of mineralized tissues and authigenic phases for fixing elements as a source of contrast between hard and soft fossil parts. Our results are based on the identification of spatial distributions of more than twenty elements in entire fossils through synchrotron X-ray spectral raster-scanning. The rationale and performance of our approach is demonstrated against three flat fossil specimens from the Late Cretaceous. To complement this elemental approach, we propose a statistical method solely based on the full XRF spectra and not depending on a model and corresponding parameters. While not providing quantitative compositional information, this method offers fast and direct morphological information and may contribute towards the chemical analysis of the sample. Using this combined approach, we demonstrate the ability to obtain critical morphological and chemical information on flat fossils for taxonomic, phylogenetic and microtaphonomic studies. We evaluate the potential of the method to become a widespread approach to discriminate fossil tissues at microscale and decipher complex features of taxonomic interest for entire flat fossils, in view of significantly improved virtual preparation for paleontological studies.

### Fossil Material

In this study three fossils originating from the Djebel Oum Tkout Lagerstätte (OT1; Kem Kem beds, Morocco; Cenomanian, *ca.* 95 Million years (Myr); [20]) were selected for their paleontological significance or as typical representatives of the state of preservation in Lagerstätten: (i) a specimen of the recently-described shrimp *Cretapenaeus berberus* Garassino, Pasini & Dutheil, 2006 [21] (MHNM-KK-OT 01a), (ii) a usual teleost fish displaying the same state of preservation than most of the actinopterygian fishes from the locality (MHNM-KK-OT 02), and (iii) a newly discovered teleost fish of unknown affinities (MHNM-KK-OT 03a). The latter is only known by a unique specimen. Unfortunately its skeleton, bearing most key phylogenetically diagnostic characters, is only partly accessible to light and electron microscopic characterization, precluding the description of this new genus and species. Neither the commonly performed manual preparation using fine needles, nor the virtual preparation using X-ray computed tomography was feasible for this specimen as: (i) most of its bones are tiny and easily breakable, so that use of needles is damageable for the fossil preservation, (ii) the clayey sedimentary matrix highly flocculates in contact to liquids preventing the use of solvents to remove the fine layer of particles covering the fossil, and (iii) state-of-the-art computed tomography under optimized conditions is hardly applicable to our fossils; contrast in the material electron density allows distinguishing most of the fossil from the sedimentary matrix, but not sufficiently to attain a fast and complete reconstruction of the fossil (*SI Appendix*, Fig. S1).

## Results and Discussion

### Major-to-trace Elemental Imaging from Full Spectral Decomposition

#### Decapod shrimp

**Figure 1:**
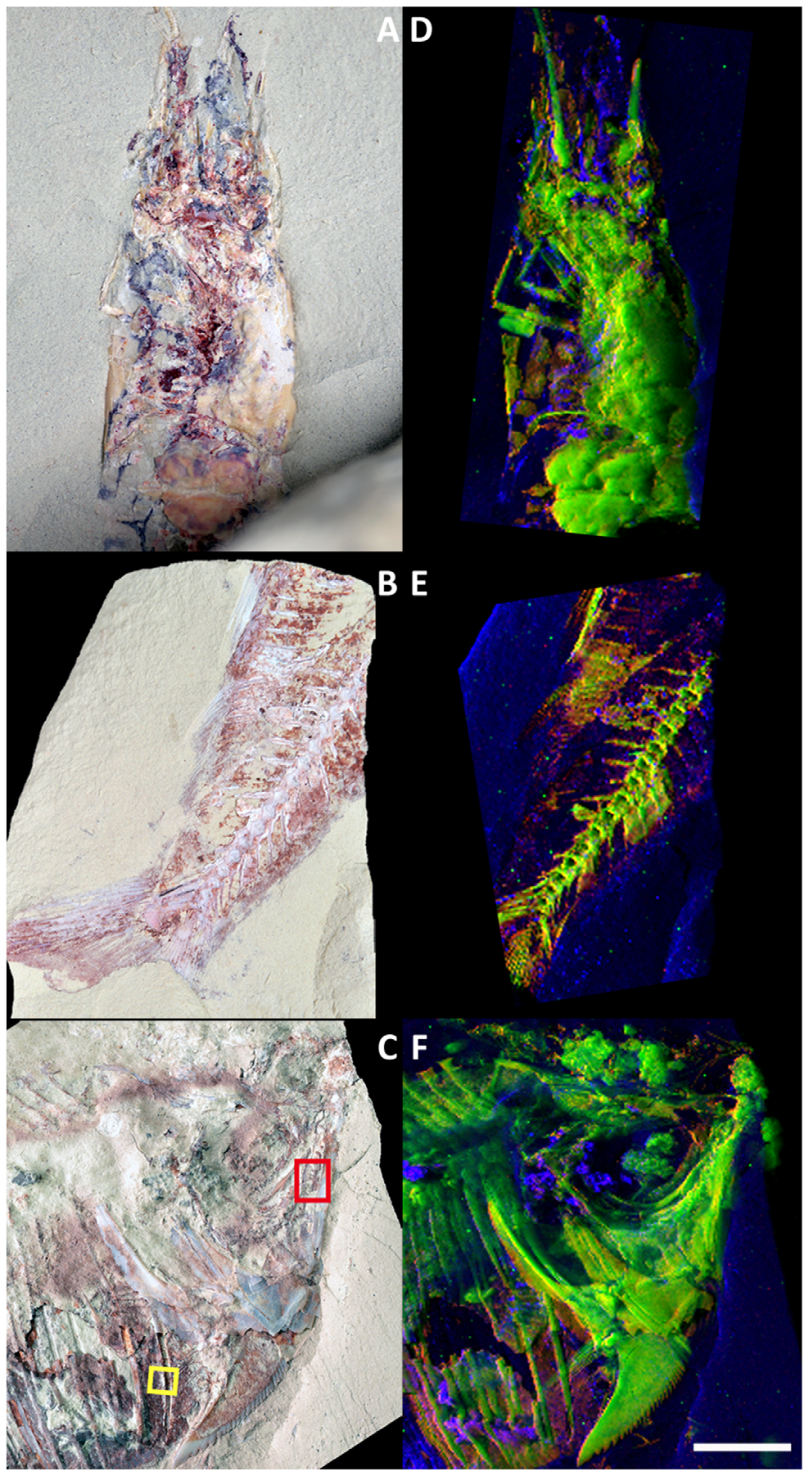
Synchrotron X-ray fluorescence mapping of major-to-trace elements in fossils from the OT1 Lagerstätte. (*A*–*C*) Optical photographs of the specimen of the shrimp *Cretapenaeus berberus* MHNM-KK-OT 01a (*A*), the usual teleost fish MHNM-KK-OT 02 (*B*) and the newly identified teleost fish MHNM-KK-OT 03a (*C*). (*D*–*F*) False color overlays of elemental distributions reconstructed from a full spectral decomposition of the synchrotron raster-scanning data. (*D*) False color overlay of neodymium (red), yttrium (green) and iron (blue) distributions from the shrimp (scan step: 100×100 µm^2^, 26,751 pixels). (*E*) False color overlay of neodymium (red), strontium (green) and iron (blue) distributions from the characteristic teleost fish (scan step: 125×123 µm^2^, 21,120 pixels). (*F*) False color overlay of neodymium (red), yttrium (green) and iron (blue) distributions from the newly identified teleost fish (scan step: 100×100 µm^2^, 50,851 pixels). Images demonstrate the strong elemental contrast between fossil skeletal and soft tissues. The yellow and red squares in *C* indicate the two areas that were mapped at higher spatial resolution in Fig. 2. The scale bar is 5 mm and applies to all panels.

*Cretapenaeus berberus* is the major component of the arthropod fauna at the OT1 locality. The investigated specimen is dorso-ventrally flattened and displays the cephalothorax, the cephalic appendages, some pereiopods and the first two pleonal somites (Fig. 1*A*). The posterior part (i.e. the pleonal somites 3 to 6, and probably telson and uropods) is embedded in the sedimentary matrix. Additional sections through the pleon were prepared to study the fossil cross-sectional composition. Both cuticular and soft-tissue remains appear phosphatized. They consist of apatite, as confirmed by X-ray diffraction and homogeneous distributions of phosphorous and calcium. Traces of strontium (typical content from processed synchrotron XRF spectrum: *ca.* 100 ppm) and yttrium (200 ppm) also characterize these tissues (Fig. 1*D*). Neodymium (0.12 wt%), lead and the highest iron contents are found in the dark-reddish areas of the specimen.

#### A characteristic teleost fish

This common fossil fish is representative of the outstanding conservation of the Lagerstätte fish fauna. This fossil shows 16 vertebrae, the caudal skeleton, the anal and pelvic fins and areas where muscles were exceptionally well-preserved (between the anal and pelvic fins areas, over the neural spines dorsally to the anal fin, and often along the haemal and neural spines; Fig. 1*B*). As major elements involved in bone mineralization (bioapatite), imaging demonstrates the dominant presence of calcium and phosphorous along the preserved structures of the fossil. The distribution of calcium highlights the fine morphological features of the skeletal organization: vertebrae, caudal skeleton and fins (Fig. 1*E*). Other chemical elements are found in minor-to-trace quantities along the fossil. In particular, the distribution of traces of strontium (150 ppm) and REEs is confined to bony material. Thick muscles, finely mineralized in apatite, are revealed by calcium, phosphorous and the REE neodymium (0.15 wt%) found in high concentrations both in bones and in mineralized muscles.

#### A newly identified teleost fish

**Figure 2:**
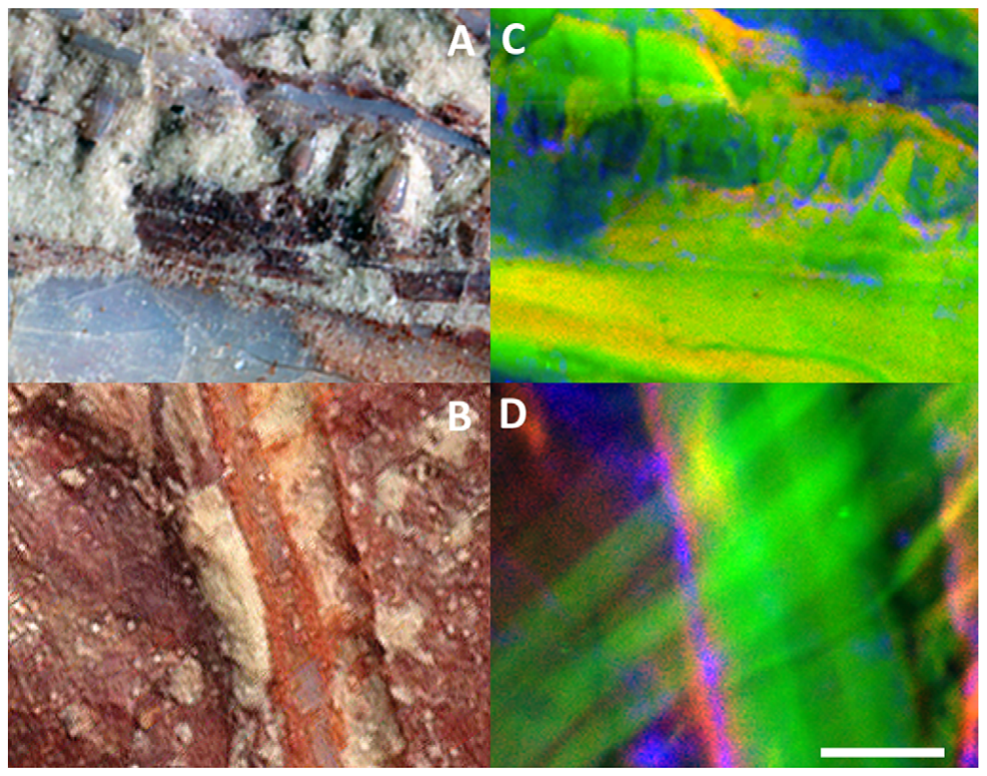
Microscale visualization of previously indiscernible anatomical details from the new teleost fish MHNM-KK-OT 03a using synchrotron XRF mapping. (*A*) Close-up of its jaws from the red square in Fig. 1*B*. (*B*) Close-up on the area from the yellow square in Fig. 1*B*. (*C*–*D*) False color overlays of elemental distributions reconstructed from a full spectral decomposition of the synchrotron data. (*C*) False color overlay of phosphorous (red), yttrium (green) and potassium (blue) distributions from the jaws area *A*, allowing the visualization of hidden teeth (scan step: 10×10 µm^2^, 28,650 pixels). (*D*) False color overlay of neodymium (red), yttrium (green) and iron (blue) distributions from the area *B*, showing hidden fin rays back to the cleithrum (scan step: 5×5 µm^2^, 32,361 pixels). The unique scale bar represents 500 µm in *A* and *C*, 50 µm in *B* and *D*.

This critical specimen for fish evolution displays a peculiar, notched, elongated bone behind the cleithrum (Fig. 1*C*). It is the only teleost ever reported to display such a peculiar bone, and therefore a yet undescribed taxon. The posterior part of the skeleton, which bears most of the key phylogenetic characters, is absent from this fossil. In order to decipher the fish taxonomic affinities, one has to rely exclusively on anatomical details from the preserved anterior part. Unfortunately, critical features, such as the neurocranium, fins, vertebrae and rib insertions remain hidden under sediment or exceptionally well-preserved, phosphatized muscles. In like manner to the previous specimen, distributions of calcium, phosphorous and minor-to-trace elements highlight the microscale morphology of the skeletal organization: skull bones and the anterior part of the axial skeleton at a medium resolution (100×100 µm^2^, Fig. 1*F*), and previously hidden teeth and fin at a higher resolution from the areas denoted by a square respectively in red and yellow in Fig. 1C (10×10 µm^2^ and 5×5 µm^2^, Fig. 2). The spatial distribution of these trace elements is essentially confined to bony material (skull bones, axial skeleton and fin rays; *SI Appendix*, Fig. S2). Particularly, traces of strontium (150 ppm) and yttrium (550 ppm) allow straightforward visualization and data segmentation of the axial skeleton and skull bones from the sedimentary matrix. Most of the elements from the lanthanide series are also found in significant quantities in bones (e.g. lanthanum: 750 ppm, neodymium: 0.18 wt%, gadolinium: 550 ppm and ytterbium: 10 ppm). Besides calcium and phosphorous, yttrium and the REEs, particularly neodymium (900 ppm), are all also found in significant quantities in the fossilized muscles.

### Visualization of Previously Indiscernible Anatomical Features and Tissular Elemental Chemistry

Non-invasive synchrotron XRF imaging allowed a description of the new fossil fish (Figs. 1*F* and 2). Elemental contrast from our approach yields the very first observation of the entire skull, together with vertebrae and rib insertions. Visualizing supplementary anatomical details was essential in order to resolve its taxonomic affinities. While light and electron microscopy studies did not allow for identification of the peculiar notched bone and of how it is articulated to other skull and girdle bones, XRF mapping clearly shows that it displays a joint with the cleithrum/scapula. It also gives anatomical and morphological information on the anatomy of other skull bones, previously hidden under a fine layer of sediment. In particular, it shows the neurocranium that extends into a sharp supraoccipital at the top of the skull, the metapterygoid, and the hyomandibular that appears dorsally flared. Such a virtual reconstruction of the whole skull was provided by the information depths from strontium and yttrium K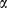 lines excited by the hard X-rays (respectively 244 and 285 µm in pure apatite with our geometry and considering a 10% attenuation) that gave access to signals from material below the sedimentary matrix covering the specimen. In contrast, the signal from lighter elements such as phosphorous and calcium primarily originates from the top tens of micrometers close to the surface. At micrometric spatial resolution (step size in raster-scanning: 10×10 µm^2^), we distinguish hidden teeth from the lower and upper jaws of the specimen (Fig. 2*C*). Imaging clearly reveals the morphology of the upper jaw, only locally visible through light and electron microscopy. This demonstrates that this fish possessed only one tooth row. It also highlights straight fin rays hidden behind the cleithrum (Fig. 2*D*, step size in raster-scanning: 5×5 µm^2^). Such a position clearly indicates the pectoral fin, and strongly helps in identification of the other fins, previously impossible although of major interest.

**Figure 3:**
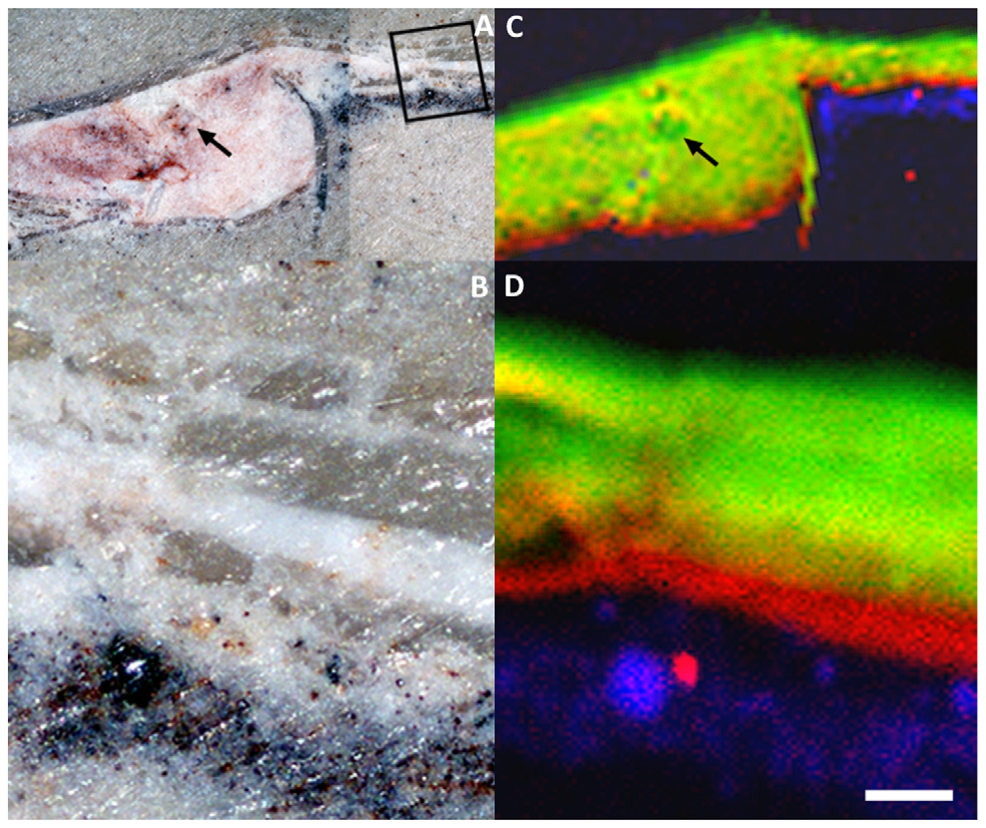
Discrimination among fossil tissues within the shrimp *Cretapenaeus berberus* MHNM-KK-OT 01a. (*A*) Optical photograph of a cross-section through the fourth somite of the shrimp. (*B*) Close-up from the pleuron located by the black square in *A*. (*C* and *D*) False color overlays of neodymium (red), yttrium (green) and iron (blue) distributions through a full spectral decomposition from *A* (scan step: 100×100 µm^2^, 13,578 pixels) and *B* (scan step: 5×5 µm^2^, 13,231 pixels) respectively. The black arrows in *A* and *C* localize the digestive tract. The unique scale bar represents 500 µm in *A* and *C*, 100 µm in *B* and *D*.

In the dorso-ventrally flattened *Cretapenaeus berberus* shrimp, synchrotron XRF mapping allowed visualization of limbs and cephalic appendages (Fig. 1*D*, see particularly the second antennae). Arthropod appendages bear key characters to resolve taxonomic affinities (*e.g.* five pairs of pereiopods on the last five pereional segments as an apomorphy of Decapoda, dicondylic metapterygotan mandibles considered as an apomorphy of the winged Metapterygota). Unfortunately they are only rarely preserved, and even in that case, their study remains challenging because fossil arthropods are often extremely flattened during fossilization and the different fossilized tissues appear superimposed. Gaining access to these appendages using such new imaging techniques is therefore of great interest to paleontologists. A cross-section through the posterior part of the shrimp pleon shows heterogeneity in REE response to display a strong contrast between cuticle and soft tissues (Fig. 3*C,D*). The dorsal and ventral cuticular elements have different REE compositions: the former with a high yttrium contribution (green color in the false color overlays), the latter with a high neodymium contribution (red color). The mineralized soft parts inside the somite exhibit a heterogeneous mix of yttrium and neodymium (yellow color). The green disc discernible in the center of the somite (high yttrium contribution, marked by the black arrow in Fig. 3*A,C*) represents a section through the digestive tract, highlighting a contrast in REE incorporation within soft-tissues.

In the characteristic teleost fish, elemental contrast notably allows distinguishing previously indiscernible haemal spines (high yttrium contribution) from “casing” well-preserved muscles (high neodymium contribution) (Fig. 1*E*).

Our approach therefore provides a direct way to study the chemistry of biomineralized tissues and to describe previously indiscernible anatomical features of fossil animals (both vertebrates and invertebrates) on the basis of chemical contrasts at high spatial resolution, with large spatial dynamics from micrometers to tens of millimeters.

### Contrasts in Elemental Composition

Elemental signatures will enhance contrast between distinct fossilized tissues if initial concentrations, sorption and/or substitution rates significantly differ from one tissue to the other. Imaging of the multielemental major-to-trace distribution particularly showed that the distribution of REEs is contrasted among fossil tissues (Figs 1, 2 and 3). Indeed, a number of past studies based on point quantification and profiling showed that yttrium and a number of REEs are present in significant quantities in fossil bones, teeth and sedimentary apatite [22]. Among fossil bones and teeth of a broad range of vertebrates from the Late Devonian to the Quaternary collected all around the world [22]–[24] and in our specimens as well, the total REE content was shown to be in the hundred ppm concentration or greater, a range straightforwardly attainable with our approach. Such amounts by far exceed that encountered *in vivo* and in modern biogenic apatites, both with REE concentrations typically in the lower ppt (parts per trillion) to ppb (parts per billion) range [25]–[27]. REEs concentration is known to increase by three to four orders of magnitude within 10^3^–10^4^ years in fossil bones through intake from the fossilization context [28].

REEs are scavenged from environmental waters by sorption and substitution within bioapatite crystals or association with detrital or authigenic phases [28]. REE distribution and fractionation depend on fossilization and diagenesis processes, leading to their use in paleontological, paleoenvironmental and taphonomic research [22], [29], [30]. Relative REE amounts reflect simultaneously the connectivity of the environmental water network, local redox, the specific surface area of the bioapatite nanocrystals and physico-chemical conditions and properties of substituted apatite. In the apatite crystal lattice, REEs and strontium substitute calcium isomorphously, as *e.g.* the ionic radius of Nd^3+^, 1.25 (CN = 8), matches within 1% to that of Ca^2+^. Substitution rates of REEs for Ca^2+^ in the bioapatite lattice are governed by differences in crystal lattice strain [31], [32]. Metastable bioapatite nanocrystals tend to dissolve and recrystallise until the bone possibly reaches a relatively close-pore state. This is a critical assumption for paleoenvironmental reconstructions and REEs were studied as proxy for fossil taphonomy, paleoenvironments and vertebrate provenance, although significant exchanges were shown to take place hundreds of thousand years after burial [22], [23], [29]. All above-mentioned environmental and physico-chemical factors will likely lead to the differences in content in the elements that substitute calcium in fossilized tissues and provide the ability to discriminate micromorphological features through trace elemental imaging.

### Discrimination among Fossil Tissues

**Figure 4:**
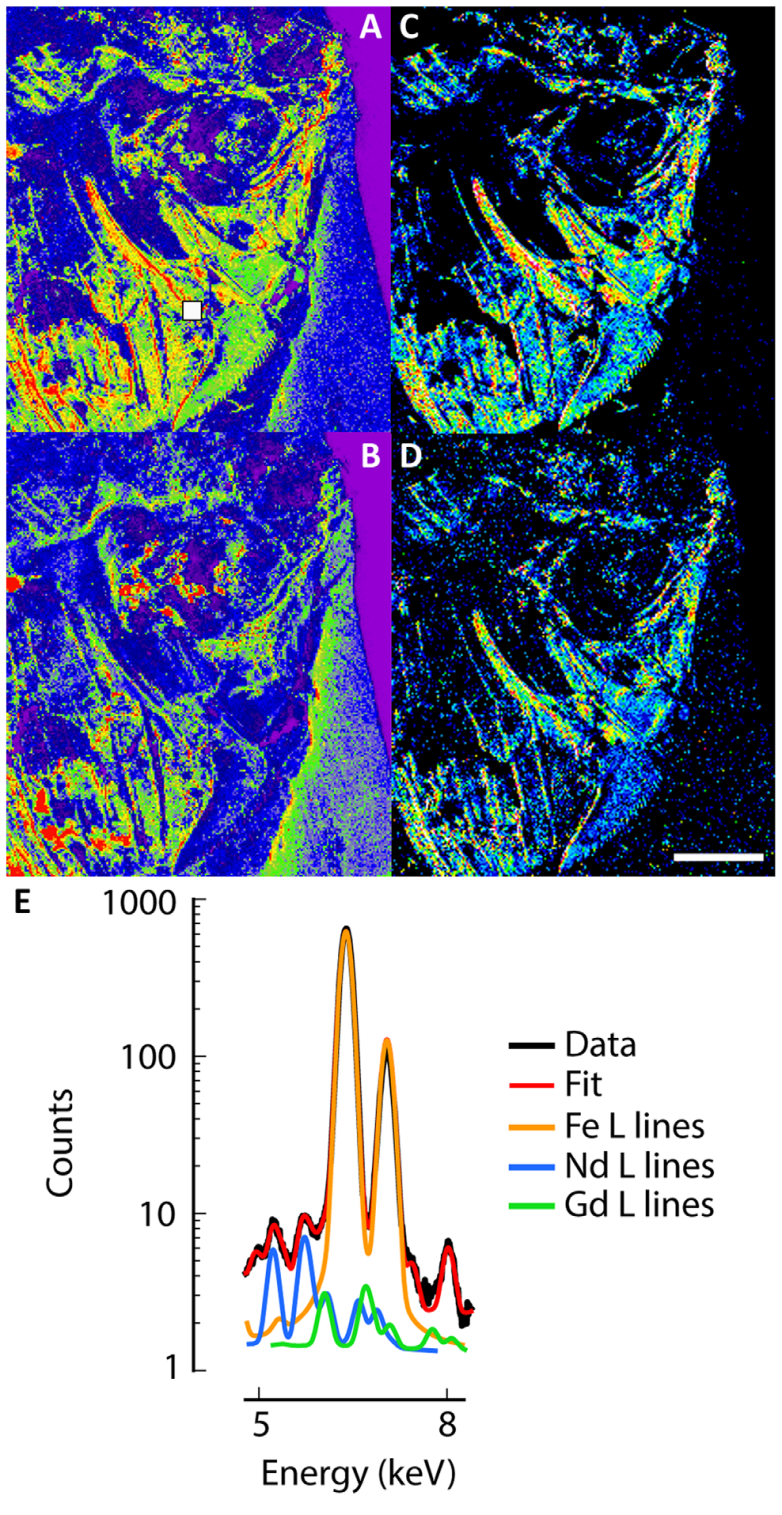
Comparison of images produced by integration of counts in regions of interest and spectral decomposition of the full spectra from the new teleost fish MHNM-KK-OT 03a. (*A* and *B*) Images from the integration of counts in regions of interest corresponding to the energy domains of neodymium (*A*) and gadolinium (*B*). (*C* and *D*) Distributions of neodymium (*C*) and gadolinium (*D*) after spectral decomposition of their respective contribution to the full spectra. (*E*) Mean XRF spectrum (black line) and fit line (red) between 5 and 8 keV from the 9×9 pixels white square area in *A*, and contributions from iron K lines (orange), neodymium L lines (blue) and gadolinium L lines (green). Total mean XRF spectrum and fit line are given in *SI Appendix*, Fig. S3. The scale bar represents 5 mm.



**Figure 5:**
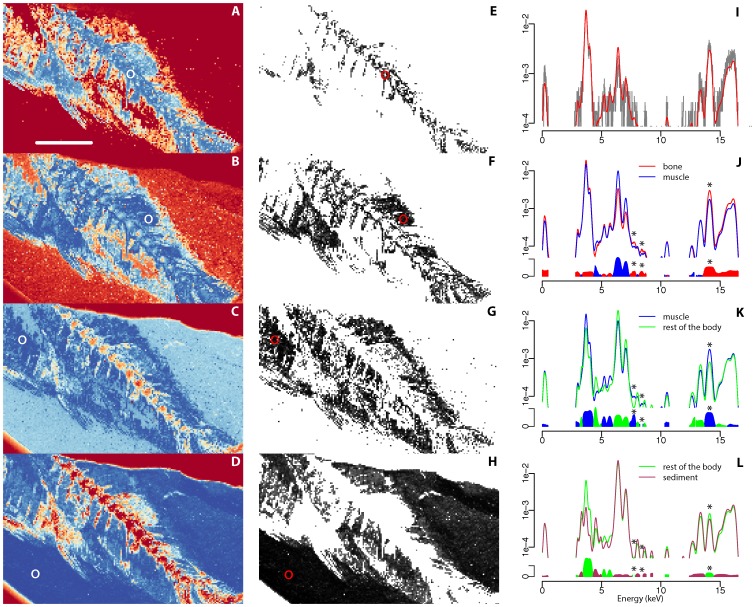
Statistical discrimination of synchrotron XRF data from distinct tissues in the characteristic teleost fish MHNM-KK-OT 02. (*A*–*D*) False color representations of the Kullback-Leibler divergence of spectral densities of each pixel from that of the pixel located at the center of the white circle, respectively characteristic of bone (*A*), muscle (*B*), rest of the body (*C*) and sedimentary matrix (*D*). The color scale goes from blue (for high similarity to the selected pixel) to red (high divergence) going through yellow. (*E*–*H*) Weighting maps for each of the selected pixels. White correspond to pixels that were ignored, grey to black are increasing weights from 0.8 to 1. (*I*) Measured spectral density (grey) and its denoised estimation (red) for the pixel characteristic of bone (*A* and *E*). (*J*–*L*) Pairwise comparisons of denoised estimates of the spectral densities for the pixels indicated on each figure: superposition of the estimated spectral densities (top) and log-ratio of the spectral densities (bottom, amplitude being the absolute log ratio, color at a given energy corresponding to the higher spectral density for this energy). Dots correspond respectively, going from low to high energy, to the L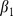 of erbium (7.811 keV), the L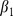 of ytterbium (8.402 keV) and K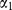 emission lines of strontium (14.165 keV). The scale bar represents 5 mm.

While elemental composition maps are essential to get the precise composition of each region of the fossil and to offer chemical information on the preservation mechanism, they are not providing direct morphological information. The derivation of morphological information from the elemental maps will always be limited by the quality of the model used in the decomposition process. The simple display of the integrated intensity in spectral regions of interest is often sufficient to show contrasts in elemental concentrations. However, when distinct X–ray emission lines of informative elements superimpose, an efficient procedure is to perform a full spectral decomposition. The distribution of trace elements can then be imaged even when their lines fall in the same energy domain than major elements. As an example, the L–lines of gadolinium overlap with iron K–lines. In our fossils, calcium and iron are major and ubiquitously–distributed elements. The full spectral decomposition of the XRF spectra reveals that gadolinium is confined to bone and mineralized soft–tissues (Fig. 4*D*). Simple peak integration at the Gd L-line leads to an artifactual image, as it is dominated by the iron signal (Fig. 4*B*). The distribution of gadolinium is confirmed from its similarity to other REEs such as neodymium, which L-lines do not overlap with those from any major element (Fig. 4*A,C,E*). Then the morphological information contained in the superimposed regions of the spectra will be lost at the modeling step and will not be recoverable during image analysis and segmentation step. The spectral decomposition process does not suffer from such superimposition but is time-consuming and sensitive to the experimental parameters. To overcome these limits we propose a method that principally targets the morphology question and does not go through a model based data reduction step. This method is designed as a statistical approach directly using the set of full-range XRF spectra. We consider each spectrum as the result of a global interaction mechanism including X-ray absorption, primary fluorescence, re-absorption as well as all higher-order mechanisms that influence this spectrum. In this framework the spectrum is the empirical estimation of the probability that a photon emitted by the material under the X-ray beam and received on the detector is of a given energy. We will refer to this distribution as the *spectral density* (eq. 1), noted *s*. For a measured spectrum *S*, the *spectral density* is estimated by
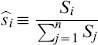
(1)where *S_i_* and 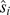 are respectively the number of photons and the estimated spectral density for channel *i*, and *n* is the total number of channels of the analyser. This probability distribution is characteristic of the material present in the photon/material interaction volume. The function that gives the spectra from the material is differentiable and surjective: two pixels with the same spectra might have different compositions but two pixels with differing spectra cannot have the same composition and stratigraphic repartition. Hence, except for a scale factor, discrimination of the spectral density is a consistent base for the morphological study of the fossil. In practical terms, differences between spectral densities are mostly due to variations of the relative concentrations rather than absolute ones, but might also be affected by the stratigraphic repartition of each constituent through variation in photon re-absorption. In order to compare the spectral densities of each pixel of the map, we used the Kullback-Leibler divergence (eq. 2) which is a natural dissimilarities measure for probability distributions:

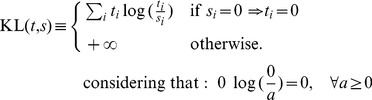
(2)We then can plot false color images of the divergence of spectral densities of each pixel from that of a preselected pixel by applying a color scale between high similarity and high divergence (Fig. 5*A*–*D*). Furthermore KL*(t,s)* is the expected log-likelihood ratio between the spectral densities *s* and *t* when *t* is considered as the true probability distribution. Hence we can use 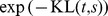 as a weight for spectral density *s* to estimate a denoised spectrum for pixels corresponding to *t* through averaging of measured spectral density, after *ad hoc* thresholding (histograms and thresholds are given in *SI Appendix*, Fig. S4).

This probabilistic approach leads to clear discrimination of the different fossilized tissues and the sedimentary matrix and provides direct morphological information. In the characteristic teleost fish we clearly discriminate bones, mineralized muscles, the rest of the body and the surrounding sedimentary matrix on the basis of variations in elemental relative concentrations (Fig. 5*E*–*H*). Particularly, it allows discriminating previously indiscernible haemal spines hidden within mineralized muscles in the ventral area of the fish (Fig. 5*F*). Besides yielding morphological information it also contributes towards the chemical analysis of the sample by providing denoised spectral densities (Fig. 5*I*) and by allowing energy domain localization of the differences between spectral densities, *i.e.* identification of the main changes in elements between distinct fossil tissues (Fig. 5*J*–*L*). Our approach therefore appears very promising to visualize critical superimposed morphological features in flattened fossils, and to get insight into the taphonomical processes.

## Conclusions

We have reported that synchrotron fast XRF raster-scanning can routinely be applied to discriminate tissues in flattened fossils, such as the vitally interesting specimens exceptionally well-preserved in Lagerstätten. A great advantage of the method is its non-destructiveness and non-invasiveness: no sample preparation is needed further than the preliminary usual cleavage of the sediment blocks. High spatial resolution, high signal-to-noise ratio and collection of full-range XRF spectra that are ideally provided at a synchrotron beamline allow performing a spectral decomposition for all constitutive elements. This is only attained when taking into account all of them, including those at trace levels. Minor and trace elements, particularly strontium, yttrium and other rare earth elements, in particular lanthanum, cerium, neodymium, samarium, gadolinium, dysprosium, erbium and ytterbium, all among the most abundant rare earths in the Earth crust, are easily mapped within entire flat fossils at microscale. Multi-elemental imaging of these elements, known to be in a straightforwardly detectable content range on fossils of more than 100 kyr old, provide critical information on the morphology of exceptionally well-preserved fossils. Since flat fossils are compressed, the typical information depth of X-ray emission lines is well adapted to their analysis. Images are produced with the flexible spatial resolution needed to resolve both millimetric and micrometric details. Using different fluorescent lines and/or different elements from the same structure allows tuning the probed depth and distinguish, in a certain measure, surface from volume. While this complete elemental reconstruction takes a long data processing time (from several hours to a few days for large maps) and remains sensitive to experimental parameters, we develop here a much faster approach based on a statistical analysis of full spectra and providing simultaneously morphological information through a clear discrimination of the different fossilized tissues, denoised spectral densities and energy domain localization of the differences between spectral densities.

XRF scanning methods are available at most 3rd generation facilities and thereby widely accessible to users. Further implementation of so-called ‘fly-scan’ modes will allow collection of full-range XRF spectra with millisecond-range counting time per pixel. Future paleontological investigation will benefit from such developments to (i) discriminate fossil tissues at microscale over large areas, (ii) infer taxonomic information from more accurate description of fossil characters, (iii) better describe fossilization and taphonomical processes, and (iv) gain paleoenvironmental information from trace elemental fractionation, including REEs, in correlation to isotopic data.

## Materials and Methods

### Material

Fossils studied herein were found in Djebel Oum Tkout (OT1, Kem Kem Beds, Morocco) in november 2012 and belong to the Natural History Museum of Marrakesh, Morocco (MHNM). They consist in MHNM-KK-OT 01a, a specimen of the recently-described shrimp *Cretapenaeus berberus* Garassino, Pasini & Dutheil, 2006 [21]; MHNM-KK-OT 02, a usual teleost fish displaying the same state of preservation than most of the actinopterygian fishes from the locality; and MHNM-KK-OT 03a, a newly discovered teleost fish of unknown affinities. All necessary permits were obtained for the described study, which complied with all relevant regulations. The Moroccan Ministère de l’Énergie, des Mines, de l’Eau et de l’Environnement delivered permits for the field compaign. This material was taken to the Muséum national d’Histoire naturelle (MNHN, Paris, France) for study and is at the moment housed there within an agreement between the MNHN and the MHNM, and will be returned to the MHNM after study. Requests for materials can be sent to njalil@ucam.ac.ma.

### The Djebel Oum Tkout Lagerstätte

Fossils from the continental Djebel Oum Tkout Lagerstätte are found in a 0.50 m thick clayey beds at the bottom of the Upper unit of the Kem Kem beds. Most of the specimens display an exceptional preservation of soft-tissues, particularly consisting in finely mineralized muscles showing the striation of the muscles fibers through SEM imaging [20]. The faunal assemblage consists of mollusks, insects, isopods, malacostracan decapods, elasmobranchs and actinopterygians; the flora is represented by gymnosperms and angiosperms [20], [21], [33]–[35]. The scarce record of freshwater deposits during the Late Cretaceous makes the Djebel Oum Tkout Lagerstätte a snapshot of paramount importance for our understanding of the evolution of Cretaceous continental ecosystems.

### Synchrotron Raster-scanning X-ray Fluorescence (XRF)

Synchrotron XRF spectral raster-scanning was performed at the DIFFABS beamline of the SOLEIL synchrotron source (Saint-Aubin, France). XRF maps were collected at an excitation energy of 17.2 keV optimized for excitation of K-lines from phosphorous to yttrium and L-lines from cadmium to lead. The X-ray beam was collimated by 2 bendable mirrors, monochromatized (ΔE/E
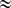
10^−4^) using a Si(111) double-crystal monochromator and focused using a Kirkpatrick-Baez mirror down to a diameter of 10×7 µm^2^ (H×V, full width at half maximum). A 500 ms counting time was used to obtain good statistics on trace elements at an energy of 17.2 keV. Photon flux at this energy is 5.10^9^ photons/s in the focused spot. The sample was mounted on a *xyz* scanner stage, allowing ± 12 mm movements with better than 500 nm accuracy. The sample is orientated at 45 to the incident beam and at 45 to the detector for XRF signal collection. The detector used was a photon-counting four elements silicon drift diod detector (SDD) for the shrimp *C. berberus* and the newly identified teleost fish (Vortex ME4, total active area: 170 mm^2^), and a mono element SDD for the characteristic teleost fish (Vortex EX, total active area: 100 mm^2^). The SDD is placed at 90 from the incident beam in the horizontal plane (plane of the electron orbit in the synchrotron). Consequently, the isotropically emitted fluorescence is recorded with strongly reduced Thomson scattering (in theory null).

### XRF Data Processing and Image Rendering

The elemental distributions presented (all except Fig. 4*A,B*) have been processed through a full spectral decomposition using the PyMca software [36] and images are displayed in full range. Semi-quantitative elemental determination was performed in the process. Identification of X-ray lines in Fig. 4*A,B* has been performed through self-written routines using the ‘R’ statistical environment [37] with the ‘SpectralImage’ package (S.X. Cohen, IPANEMA) that will soon be openly distributed through CRAN. For display, for each elemental image, the lowest value of the 2nd percentile and the highest value of the 98th percentile of the intensity distribution were used as end points for an intensity stretch.

### Statistical Discrimination of Tissues

The use of the Kullback-Leibler divergence as both a dissimilarity measure and base for a weighting scheme is hindered by the empirical and discrete nature of the t and s probability distributions: 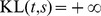 when 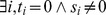, even though the rest of the probabilities fits perfectly. Since these probability distributions are empirical estimates rather than the true ones, it does happen that 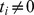 and 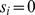 for values of *i* for which neither true probability are null. In order to avoid this issue, and given that the raw spectral signal is the discrete number of photons within a channel of the detector, 1 photon is added to each channel of *s* before computing the KL terms.

If we note *n* the number of channels used for the spectra, *S_i_* and *T_i_* the number of photons counted on channel *i* respectively for the *evaluated* and *reference* pixels (denoted pixels 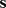 and 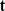) and the corresponding empirical distributions 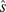 and 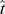 (defined in eq. 1 in the article), then we estimate KL through:
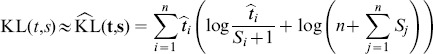
(3)


Given a *reference* pixel 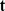 we can then plot a similarity image, displaying for each pixel 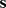 of the image *I* the value of 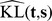. We can also compute an *estimated denoised* spectral density 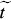 for pixel 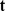. Since 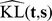 is bounded, even the most diverging spectra have a low divergence, causing a non-insignificant estimated likelihood. Hence we have to apply a threshold (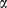), keeping in the weighted averaging only spectra which are close to the reference, so that the denoised spectral density is not affected by the very large number of pixels that have a low likelihood:
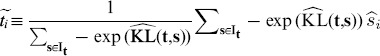
(4)with 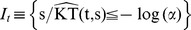 being the set of pixels that have a likelihood value above the threshold.

### Micro-computed Tomography (CT) Scanning

µCT scanning was conducted at the X-ray Tomography Imaging Platform AST-RX of the MNHN, using a GE Sensing and an Inspection Technologies phoenix| x-ray v| tome|×L240-180 CT scanner. The scan was made with an isotropic voxel size of 24.71 µm under a voltage of 83 kV and a current of 250 µA. 2000 projections over 360 degrees with 500 milliseconds of exposure time were used, with 3 averaged images per projection and 1 skipped image before each projection. Images were reconstructed using the phoenix datos| xfi 2.0 reconstruction software, and exported into a 16 bits TIFF image stack constituted of 1496 slices. Post-processing was realized at the Paleontology Imaging Unit of the MNHN Département Histoire de la Terre/CNRS UMR 7207 using VGStudioMax (Volume Graphics).

## Supporting Information

Figure S1 **Micro-computed tomography scanning of the new teleost fish.** (*A* and *B*) 3D rendering of the fossil within (*A*) and isolated from (*B*) the sedimentary matrix after rapid segmentation. (*C*) Microtomographic slice through the fossil. The contrast is sufficient to reconstruct most of the fossil but not to perform a fast complete reconstruction. A fully detailed reconstruction requires manual segmentation slice by slice of the reconstructed volume by a specialist of the anatomy of the fossil under investigation. Such a meticulous work typically requires several weeks to a couple of months, without being sure to attain a reconstruction as useful as the one obtained with the proposed methodology. Note that high absorption from metal-rich spherules prevents a clear segmentation of large areas at the back and the top of the skull. Voxel size: (24.7 µm)^3^. The scale bars represent 5 mm.
(TIF)

Figure S2
**Optical photograph of the newly identified teleost fossil fish and P, K, Ca, V, Mn, Fe, Ni, Cu, Zn, Ga, Br, Rb, Sr, Y, the REEs La, Ce, Nd, Sm, Gd, Dy and Yb, and Pb XRF maps from the yellow square area after spectral decomposition.** Scan step: 100×100 µm^2^, 50,400 pixels. Information depth in pure apatite Ca_10_ (PO_4_)_6_ (OH)_2_ from a 10% attenuation length at 45 collection: for K (K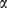: 3.31 keV) 13.7 µm; Ca (K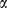: 3.69) 18.4 µm; Ti (K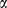: 4.51) 10.1 µm; V (K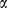: 4.95) 13.0 µm; Fe (K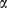: 6.39) 26.1 µm; Rb (K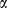: 13.35) 208.4 µm; Sr (K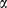: 14.16) 243.7 µm; Y (K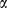: 14.96) 285.0 µm; Nd (L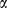: 5.22) 15.0 µm; Pb (L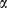: 10.50) 104.9 µm.(TIF)

Figure S3 **Mean XRF spectrum and fit line from the 9**×**9 pixels area taken from the cleithrum of the newly identified fish MHNM-KK-OT 03a shown in** **Fig. 4** **.** A good correlation between the fit (red line) and the experimental spectrum (black line), as illustrated here, is required for spectral decomposition.
(TIF)

Figure S4 **Histograms obtained from conversion of the Kullback-Leibler divergence into a likelihood in the characteristic teleost fish MHNM-KK-OT 02.** This figure is a complement to Fig. 5 that shows the histograms used to determine the threshold to produce Fig. 5*E*–*H*. (*A*–*D*) False color representations of the Kullback-Leibler divergence of spectral densities of each pixel from that of the pixel located at the center of the white circle, respectively characteristic of bone (*A*), muscle (*B*), rest of the body (*C*) and sedimentary matrix (*D*). The color scale goes from blue (for high similarity to the selected pixel) to red (high divergence) going through yellow. (*E*–*H*) Histograms of the values taken by all pixels in the map given the same reference pixels after conversion of the Kullback-Leibler divergence into a likelihood. The red vertical line at 0.8 represents the threshold above which pixels were used to produce the averaged spectral density shown in Fig. 5. The scale bar represents 5 mm.
(TIF)
