# Supplementary figures and images for: Trace Elemental Imaging of Rare Earth Elements Discriminates Tissues at Microscale in Flat Fossils
Source: PLoS One. 2014 Jan 29;9(1):e86946. doi: 10.1371/journal.pone.0086946 (PMC3906100; doi:10.1371/journal.pone.0086946)

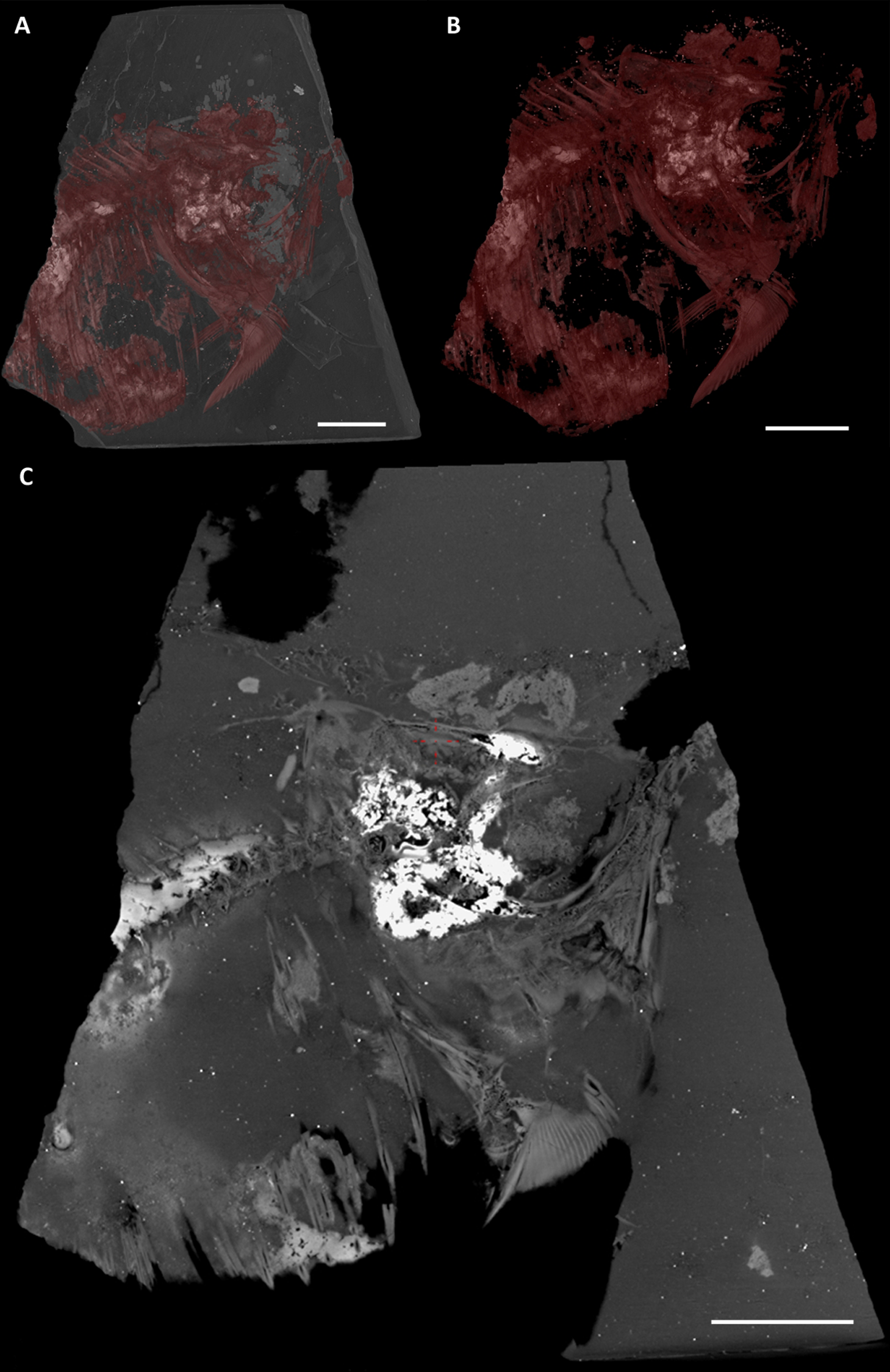

Supplement: Figure S1 — Micro-computed tomography scanning of the new teleost fish. (A and B) 3D rendering of the fossil within (A) and isolated from (B) the sedimentary matrix after rapid segmentation. (C) Microtomographic slice through the fossil. The contrast is sufficient to reconstruct most of the fossil but not to perform a fast complete reconstruction. A fully detailed reconstruction requires manual segmentation slice by slice of the reconstructed volume by a specialist of the anatomy of the fossil under investigation. Such a meticulous work typically requires several weeks to a couple of months, without being sure to attain a reconstruction as useful as the one obtained with the proposed methodology. Note that high absorption from metal-rich spherules prevents a clear segmentation of large areas at the back and the top of the skull. Voxel size: (24.7 µm)3. The scale bars represent 5 mm. (TIF) [file pone.0086946.s001.tif]

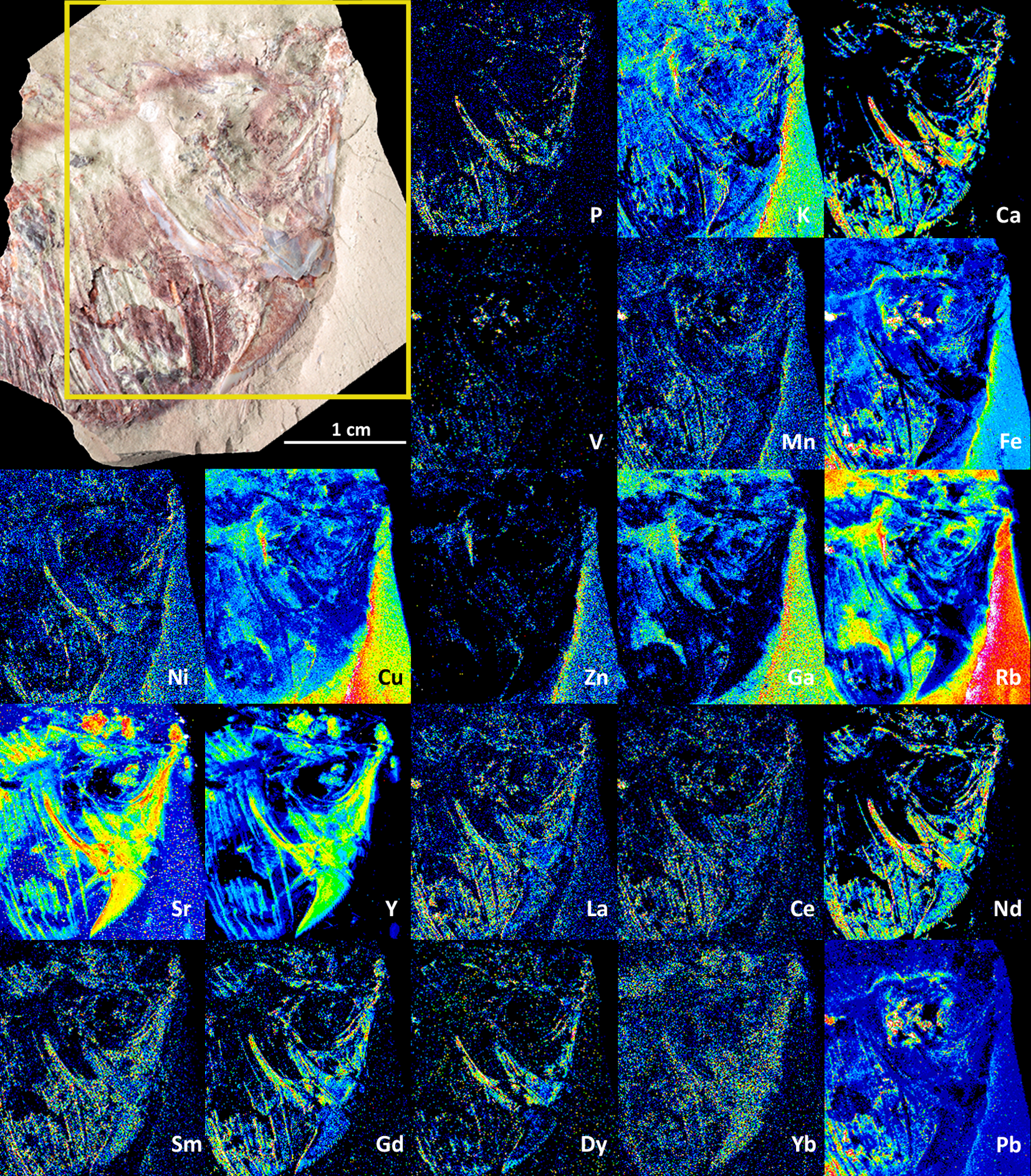

Supplement: Figure S2 — Optical photograph of the newly identified teleost fossil fish and P, K, Ca, V, Mn, Fe, Ni, Cu, Zn, Ga, Br, Rb, Sr, Y, the REEs La, Ce, Nd, Sm, Gd, Dy and Yb, and Pb XRF maps from the yellow square area after spectral decomposition. Scan step: 100×100 µm2, 50,400 pixels. Information depth in pure apatite Ca10 (PO4)6 (OH)2 from a 10% attenuation length at 45 collection: for K (K: 3.31 keV) 13.7 µm; Ca (K: 3.69) 18.4 µm; Ti (K: 4.51) 10.1 µm; V (K: 4.95) 13.0 µm; Fe (K: 6.39) 26.1 µm; Rb (K: 13.35) 208.4 µm; Sr (K: 14.16) 243.7 µm; Y (K: 14.96) 285.0 µm; Nd (L: 5.22) 15.0 µm; Pb (L: 10.50) 104.9 µm. (TIF) [file pone.0086946.s002.tif]

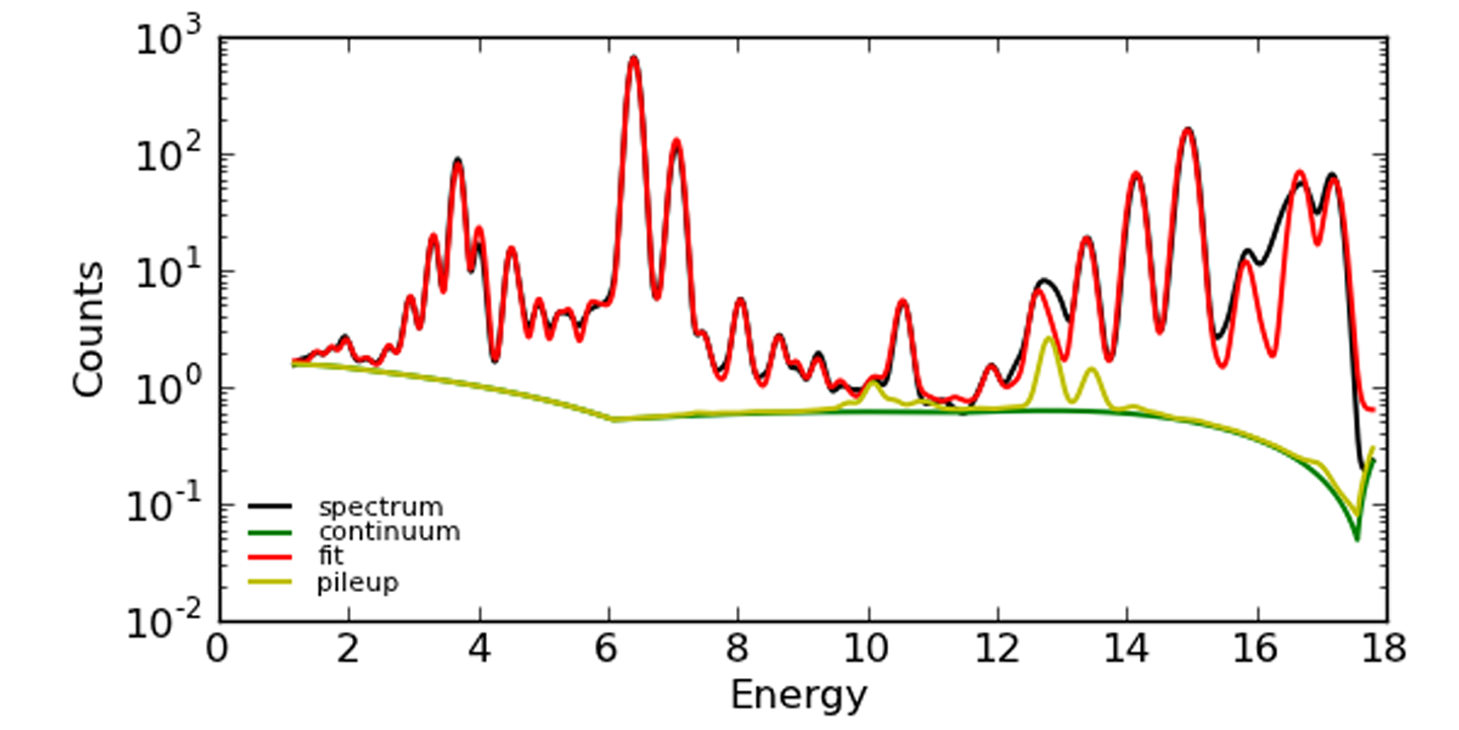

Supplement: Figure S3 — Mean XRF spectrum and fit line from the 9×9 pixels area taken from the cleithrum of the newly identified fish MHNM-KK-OT 03a shown in Fig. 4 . A good correlation between the fit (red line) and the experimental spectrum (black line), as illustrated here, is required for spectral decomposition. (TIF) [file pone.0086946.s003.tif]

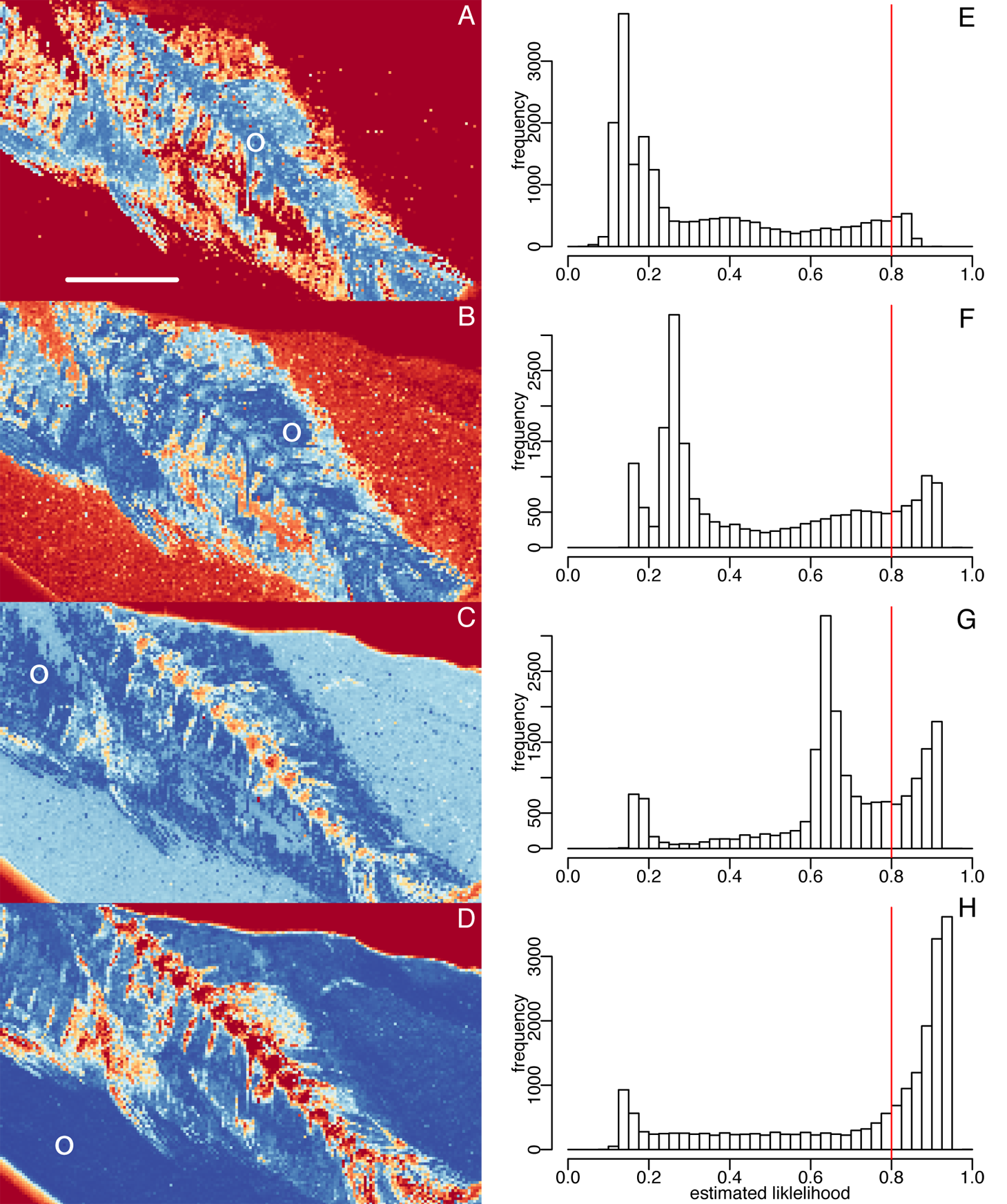

Supplement: Figure S4 — Histograms obtained from conversion of the Kullback-Leibler divergence into a likelihood in the characteristic teleost fish MHNM-KK-OT 02. This figure is a complement to Fig. 5 that shows the histograms used to determine the threshold to produce Fig. 5E–H . (A–D) False color representations of the Kullback-Leibler divergence of spectral densities of each pixel from that of the pixel located at the center of the white circle, respectively characteristic of bone (A), muscle (B), rest of the body (C) and sedimentary matrix (D). The color scale goes from blue (for high similarity to the selected pixel) to red (high divergence) going through yellow. (E–H) Histograms of the values taken by all pixels in the map given the same reference pixels after conversion of the Kullback-Leibler divergence into a likelihood. The red vertical line at 0.8 represents the threshold above which pixels were used to produce the averaged spectral density shown in Fig. 5. The scale bar represents 5 mm. (TIF) [file pone.0086946.s004.tif]
